# Who Pays for Health Care in China? The Case of Heilongjiang Province

**DOI:** 10.1371/journal.pone.0108867

**Published:** 2014-10-01

**Authors:** Mingsheng Chen, Yuxin Zhao, Lei Si

**Affiliations:** 1 School of Health Policy & Management, Nanjing Medical University, Nanjing, China; 2 Cooperative Health Humanity Initiative, Nanjing Medical University, Nanjing, China; 3 National Health Development Research Center, Ministry of Health of the People’s Republic of China, Beijing, China; 4 Menzies Research Institute Tasmania, University of Tasmania, Hobart, Australia; 5 School of Health Administration, Anhui Medical University, Hefei, China; National Institute for Viral Disease Control and Prevention, CDC, China

## Abstract

**Background:**

Health spending by the Chinese government has declined and traditional social health insurance collapsed after economic reforms in the early 1980s; accordingly, the low-income population is exposed to potentially significant healthcare costs. Financing an equitable healthcare system represents a major policy objective in China’s current healthcare reform efforts. The current research presents an examination of the distribution of healthcare financing in a north-eastern Chinese province to compare equity status between urban and rural areas at two different times.

**Methods:**

To analyze the progressivity of healthcare financing in terms of ability-to-pay, the Kakwani index was used to assess four healthcare financing channels: general taxes, social and commercial health insurance, and out-of-pocket payments. Two rounds of surveys were conducted in 2003 (11,572 individuals in 3841 households) and 2008 (15,817 individuals in 5530 households). Household socioeconomic status, healthcare payment, and utilization information were recorded using household interviews.

**Results:**

China’s healthcare financing equity is unsound. Kakwani indices for general taxation were -0.0212 (urban) and -0.0297 (rural) in 2002, and -0.0097 (urban) and -0.0112 (rural) in 2007. Social health insurance coverage has expanded, however different financing distributions were found with respect to urban (0.0969 in 2002 vs. 0.0984 in 2007) and rural (0.0283 in 2002 vs. -0.3119 in 2007) areas. While progressivity of out-of-pocket payments decreased in both areas, the equity of financing was found to have improved among poorer respondents.

**Conclusions:**

Overall, China’s healthcare financing distribution is unequal. Given the inequity of general taxes, decreasing the proportion of indirect taxes would considerably improve healthcare financing equity. Financial contribution mechanisms to social health insurance are equally significant to coverage extension. The use of flat rate contributions for healthcare funding places a disproportionate pressure upon the poor. Out-of-pocket payments have become equitable, but progressivity has decreased.

## Introduction

Financial equity in health sector reform is receiving increased attention in numerous regions [1], [2]; however, several significant challenges remain for policy-makers within this sector, for example, the question of improved equity and the associated mechanisms.

Equitable financing plays a significant role within healthcare systems for the effective provision of necessary services to all individuals. Moreover, it seeks to ensure that service provision does not expose the user to undue financial hardship, particularly with respect to poor and vulnerable sub-populations [3], [4]. Accordingly, improvement of equity within healthcare financing represents a primary policy objective in the reform and development of many healthcare systems [5], [6]. As a result, financial equity evaluation has become a fundamental process in the assessment of healthcare reform efficacy, comprising an examination of existing flaws in healthcare financing channels, in concurrence with locating effective countermeasures for the remediation of highlighted deficiencies. Healthcare financing distribution has been examined at both international (equity performance comparisons across countries) [7], [8] and national levels (equity status within different socioeconomic groups) [9].

Due to influential social and economic shifts from the early 1980s onwards, China’s healthcare system was subsequently reformed to evolve from a planned economy to a market-oriented model [10]. As a result of healthcare financing decentralization, government spending with respect to the healthcare system has declined rapidly since the initiation of these reforms. Accordingly, public funding of the healthcare system has decreased, in accord with an increased proportion of associated private financing [11]. For example, China’s social health insurance schema had traditionally consisted of the Government Welfare Insurance Scheme (GWIS), the Labor Insurance Scheme (LIS) in urban areas and the Cooperative Medical Scheme (CMS) in rural areas. GWIS primarily encompassed civil servants and other government employees, veterans, and college students; whereas LIS comprised workers and their dependents across all formal economic sectors [12], with the majority of associated medical expenditure being government funded. These schemes had faced considerable challenges brought about by market-oriented economic reforms, thus leading to major modifications with respect to hospital management procedures and financing models. Furthermore, the aforementioned reforms, coupled with the adoption of increasingly advanced medical technologies and economic inflation, had become a primary driver of increasing healthcare costs. In conjunction with increasing healthcare quality demands, and correspondingly high fiscal pressures, financing from both the GWIS and LIS have decreased significantly, thus leading to markedly higher out-of-pocket (OOP) medical expenses being forced upon individuals and households.

Prior to economic reforms, the CMS played a key role in ensuring access to basic healthcare services among the majority of the rural population, particularly the poorer sub-population [13]. This scheme was typically operated and financed by the local community, with all consumers required to pay the same premium. However, a collapse of the scheme was initiated upon implementation of China’s Household Contract Responsibility System in the early 1980s, resulting in the requirement that healthcare be funded at the household level. By 2003, just 9.3% of rural farmers were still enrolled in the CMS, with almost 80% not in possession of any health insurance coverage [14]. In the absence of coverage, farmers were required to pay for necessary health services via direct payment, thus creating significant barriers to accessing basic health services, particularly within poor and vulnerable sub-populations, in light of increasingly expensive health services and procedures [15].

The aforesaid changes have transformed China’s healthcare finance structure; between 1980 and 2002, government healthcare spending dramatically decreased from 36.24% to 15.69%, while social health insurance outlay dropped from 42.57% to 15.64%. Conversely, the share of OOP payments during the same period has soared from 21.19% to 57.72% [16]. This increasingly weighty dependence on OOP payments has resulted in the development of a socioeconomically tiered healthcare financing system, with poor and vulnerable sub-populations facing major financial difficulties when accessing healthcare services.

Results from China’s 2003 national health services survey in Heilongjiang province, the area of interest within the current study, indicate that 61.30% of individuals who should have received outpatient care (64.87% and 57.10% in urban and rural areas, respectively) did not attend any healthcare facility. Among those who were admitted but did not use inpatient services, 48.08% could not afford the associated charges [17].

This interception of increased healthcare inequalities with rapid healthcare cost escalations now represent a major crisis within the sector [18].

As previously outlined, achievement of financing equity is the primary policy objective pertaining to China’s healthcare sector reform. Since 2003, central authorities have gradually developed and implemented various reforms and policy packages [19]. A nationwide effort was initiated by China’s State Council to reform existing GWIS and LIS in urban regions. Within the region of interest in the current study (Heilongjiang province), a basic health insurance program, the Urban Worker’s Basic Medical Insurance (UWBMI), covering 26.53% of workers in 2002 and 62.01% in 2007, has been implemented within all urban conurbations [20]. Likewise, the Urban Resident’s Basic Medical Insurance (URBMI) was initiated in 2007 to provide cover for the unemployed, children, students, and elderly persons without pensions [21]. The UWBMI is jointly financed by employers and employees. The employees contribute approximately 2% of their salaries, with employers contributing 6–8% of the employee’s salaries [22]. The URBMI is funded by individuals with appropriate subsidies granted by government [23]. In categorically rural areas, the New Rural Cooperative Medical Scheme (NCMS) was initiated in 2003 to re-establish rural health insurance and further develop the rural healthcare system after the aforementioned collapse of CMS [24]. While the CMS operated at a community level and was funded jointly by locals and their associated committee, the NCMS is managed at a county level and funded by equal contributions from all enrollees and increasing fiscal inputs from both central and local authorities. NCMS is a voluntary scheme, only providing cover to enrollees; however, it has managed to provide an increasingly high level of coverage within Heilongjiang province, increasing from 51.99% in 2005 to 92.07% in 2007 [25]. Both premium and reimbursement rates have shown steady annual growth. Generally, NCMS coinsurance has exhibited a steady decrease, with covered medical services increasing on a yearly basis. Thus, social health insurance presently consists of UWBMI and URBMI in urban areas, and NCMS in rural areas. Additionally, healthcare services may be financed via voluntarily purchased commercial health insurance in market-directed economic periods; at present, this potential finance route does not represent a significant proportion of China’s healthcare financing. For example, within Heilongjiang province, commercial health insurance has accounted for approximately 4% of all financing during the last decade [16]. Healthcare financing share in different sources in Heilongjiang province between 2002 and 2007 is presented in Table 1.

**Table 1:** Share of healthcare financing amounts in Heilongjiang province during 2002 and 2007.

Year	General Taxes (%)		Social Health Insurance (%)			Commercial HealthInsurance (%)	OOP (%)
	Direct Tax	Indirect Tax	CMS(NCMS)	UWBMI	URBMI		
**2002**	3.94	11.21	1.52	15.35	\	2.25	65.73
**2003**	3.47	12.73	0.57	16.47	\	4.24	62.52
**2004**	3.07	10.11	0.91	20.99	\	4.55	60.38
**2005**	3.67	10.86	1.17	19.90	\	4.68	59.72
**2006**	4.47	12.89	2.68	21.90	\	5.36	52.69
**2007**	4.72	12.69	4.13	21.35	0.41	4.04	52.66

Data source: China National Health Accounts Report.

The outlined initiatives and measures have effectively expanded health coverage to insured individuals, in addition to encouraging the use of progressive pre-payment over direct payment, with an overall aim of reducing OOP payments and improving health financing equity. However, to date, few empirical studies have sought to provide additional evidence of the actual degree of inequality associated with China’s healthcare financing mechanisms; accordingly, an accurate assessment of the pre- and post-reform effects on the healthcare financing system is difficult to undertake. Moreover, temporal and regional financing disparities have not been adequately reviewed; these would serve to clarify both the positive and negative effects of reform driven healthcare financing. Furthermore, the identification of inherent financing flaws has been problematic, as these practices currently exist under the auspices of “hoped-for healthcare reform”. Elucidation of the issues would aid both policy makers and researchers in their assessment of financing distribution as China’s healthcare reforms evolve.

The remainder of the paper is organized as follows: Method Section describes the data and method to assess health care financing; more specifically, it outlines how empirical results in different areas and times are compared. Data about socioeconomic and health status from the national health survey on Heilongjiang province in China are then critically analyzed and evaluated. The final section discusses the results and attempts to draw some conclusions in relation to broad lessons from the Chinese experience.

## Methods

### Ethics statement

The current study was approved by the Academic Research Ethics Committee of Nanjing Medical University. As >25,000 individuals were interviewed during the study period, including illiterate and older persons, verbal informed consent was obtained prior to the commencement of interviews. Upon sampling, all interviewee names within a single community were recorded. Prior to interviewing, prospective respondents provided verbal informed consent after trained data collectors had clearly outlined both consent form and study objectives. With the permission of the respondents, data collectors marked off their name in the presence of both the respondents and supervisor. Consequently, the Academic Research Ethics Committee waived the requirement for written informed consent from these participants.

### Data sources

Data were obtained using two rounds of household surveys in Heilongjiang province, Northeast China. Surveys were conducted during 2003 and 2008 to collect data pertaining to 2002 and 2007, respectively. Heilongjiang, with a population >20 million, is considered a middle-income province in terms of per capita GDP [26]. Overall, 13 cities and counties were selected using multistage stratified random sampling; within each selected city or county, 8 communities/villages were chosen for surveying based upon economic status and geographic distribution. Subsequently, 33 households were randomly selected from each community or village; each household member was interviewed by trained data collectors. Summarily, the survey was completed by 3841 householders representing 11,572 individuals in 2003, and 5530 householders representing 15,817 individuals in 2008. Prior permission was obtained from all respondents. Data pertaining to the descriptive and socioeconomic characteristics of each income quintile are presented in Table 2.

**Table 2:** Descriptive statistics and socioeconomic characteristics of survey respondents characterized by income quintile.

Year	Income quintiles	No. ofrespondents		Annual householdexpenditure a ^,^ b		OOP		Coverage	
		urban	rural	urban	rural	urban	rural	urban	rural
2002	Q_1_	1053	1263	3832.03	3341.69	470.60	407.50	18.89%	5.04%
				(1307.11) c	(1160.81)	(405.38)	(378.30)		
	Q_2_	1054	1263	6561.71	5308.83	803.95	619.71	30.76%	7.85%
				(1664.83)	(1364.31)	(849.54)	(616.36)		
	Q_3_	1053	1257	9417.99	7096.43	1259.86	951.71	36.42%	9.14%
				(2784.26)	(1894.80)	(1214.83)	(1028.72)		
	Q_4_	1053	1264	14764.60	9571.43	1906.10	1253.91	48.61%	12.87%
				(4276.81)	(2497.10)	(1773.37)	(1378.72)		
	Q_5_	1052	1260	39202.60	16566.01	6894.29	2578.85	52.96%	13.92%
				(31196.86)	(8077.77)	(13355.73)	(5539.28)		
	Total	5265	6307	14748.75	8374.30	2308.19	1169.78	37.51%	9.75%
				(19037.86)	(6047.53)	(3259.71)	(2714.90)		
2007	Q_1_	1238	1926	5930.19	5893.59	689.73	714.09	26.79%	84.96%
				(2082.80)	(2185.30)	(700.42)	(666.42)		
	Q_2_	1237	1926	9000.18	8884.12	1283.76	1119.87	36.86%	89.19%
				(2252.93)	(2468.98)	(1237.10)	(1050.22)		
	Q_3_	1240	1925	11754.78	11443.14	1651.11	1357.52	45.07%	89.66%
				(3180.11)	(2833.74)	(1668.04)	(1337.96)		
	Q_4_	1236	1925	16082.89	14577.44	2514.17	1682.07	49.59%	89.23%
				(4452.67)	(3805.15)	(2769.66)	(1773.41)		
	Q_5_	1237	1927	31892.60	24825.89	3894.25	3486.94	69.74%	93.64%
				(21154.08)	(13833.77)	(6171.41)	(7104.05)		
	Total	6188	9629	14928.99	13122.92	2006.46	1671.82	45.96%	89.34%
				(13420.30)	(9342.74)	(3365.66)	(3505.95)		

Data source: author’s calculations based on household survey data.

a All expenditures are presented in RMB.

b All 2002 nominal prices have been adjusted to real prices from 2007 according to China’s Consumer Price Index (CPI).

c Standard deviation.

The survey was used to obtain data regarding household socioeconomic and demographic characteristics, including: household expenditure, urban–rural classification, number, gender, age and educational status of household members, the work status of household members, and household goods. In reference to household expenditure, monthly expenditure on food, traffic, housing, communication, clothing, electricity, water, fuel, education, entertainment, travel, medical care and other expenditures were recorded. Information on unexpected expenditure during the previous year was also recorded.

Healthcare expenditure was evaluated via two data sources: 1) the survey, and 2) tariffs, taxes, and copayments for social health insurance, obtained from local statistics yearbook. Specific taxes considered included taxation relating to the purchase of cigarette/tobacco, alcohol, entertainment, electricity and gas, in addition to excises associated with eating, drinking, lodging and other consumption-based taxes. Taxes were approximated via application of specific tax-rates to corresponding expenditure categories. With regard to social health insurance, flat-rate contribution was directly recorded in household interview for those respondents covered by URBMI, CMS and NCMS. Contribution was estimated for respondents covered by UWBMI via application of contribution rates to earnings of workers within the surveyed household. Commercial health insurance payments were directly obtained via surveying. Data pertaining to previous OOP payments were also recorded; these included healthcare expenditure relating to prescriptions and outpatient services paid by individuals during the 2 weeks prior to interview. Inpatient OOP expenditure were recorded for the 12-month period prior to interview, as were data relating to outpatient visits and hospital stays.

### Statistical analysis

**Figure 1:**
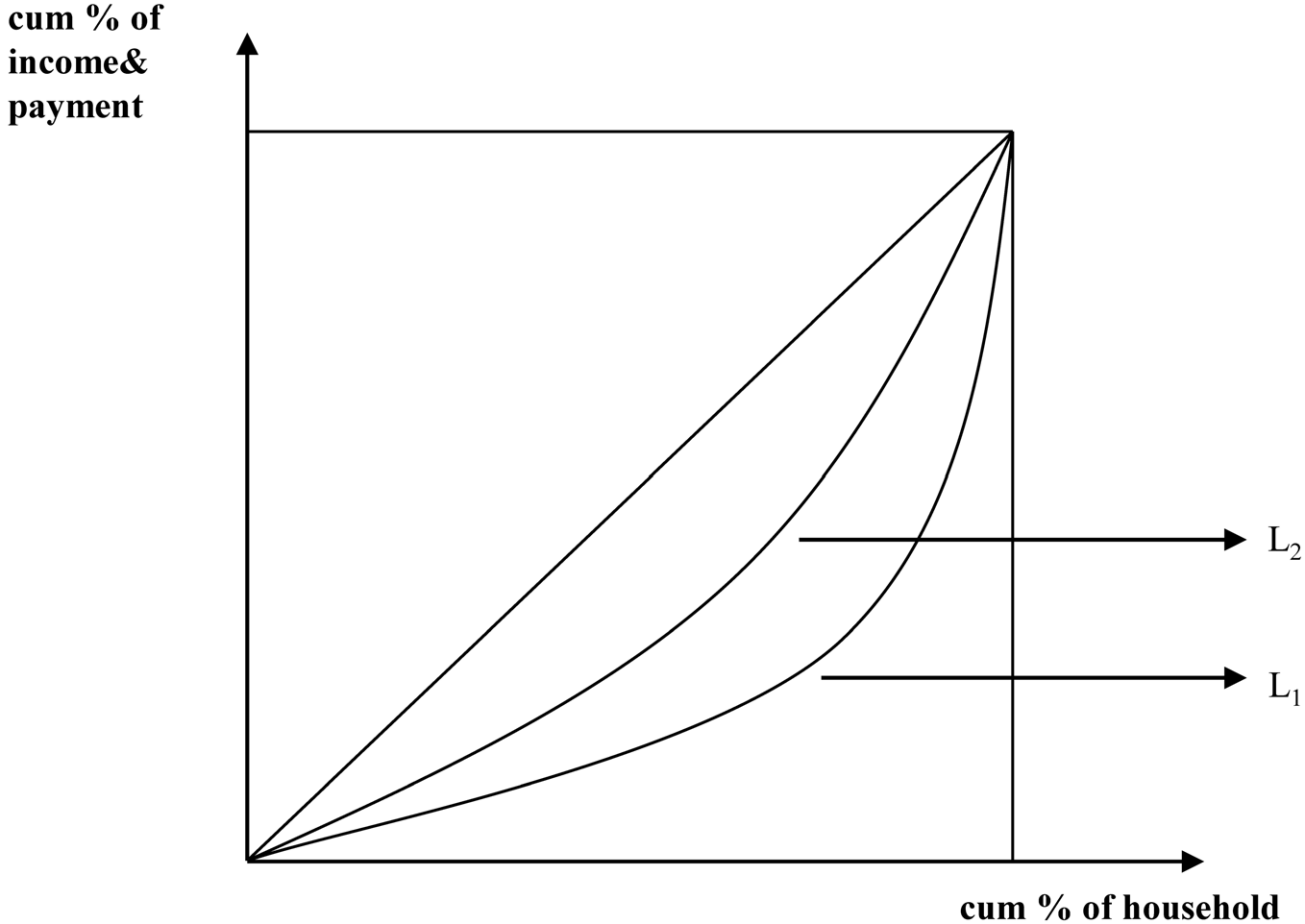
Conceptual cumulative concentration curve for healthcare payments and Lorenz curves. Conceptual cumulative concentration curve for healthcare payments and per capita household expenditure are shown. The concentration curve plots the cumulative percentage of healthcare payments (y-axis) against the cumulative percentage of the population (x-axis). Population is ranked according to ATP, from poorest to richest. The value of the concentration index is measured as twice the area between the concentration curve, L_1_, and the line of equality, L_e_ (45°line running from the bottom-left corner to the top-right). The Lorenz curve (L_2_) represents the relationship between the cumulative percentage of per capita household expenditures and the cumulative percentage of the population, which is indicated by the Gini coefficient.

While numerous approaches exist for the assessment of health financing equity, progressivity analysis has been used for the current study. Healthcare payments primarily comprise general government revenues for health and social health programs, commercial insurance programs, in addition to OOP payments. The most direct means of assessing the progressivity of health payments is via examination of the share of ability to pay (ATP) as the latter varies. Specifically, progressivity analysis measures the level of departure from proportionality with respect to healthcare payments and ATP. As shown (Figure 1), progressivity analysis compares the concentration curve for healthcare payments (L_1_) and the Lorenz curve for ATP (L_2_). Where the proportion of healthcare payments remains static with respect to ATP, the share of healthcare payments contributed by any group must correspond to its share of ATP. Under a progressive healthcare financing system, the share of healthcare payments contributed by the poor will be less than their share of ATP [27]. Thus, the Lorenz curve will be located above the concentration curve, with the opposite being true within a categorically regressive system.

The employed measure of healthcare financing progressivity was the household unit, with expenditures and healthcare payments aggregated to the household level. The degree of household expenditure was used as the measurement of ATP [27]. Adjustments were made for the size and age structure of the household via application of an equivalence scale to both ATP and each component of health financing, as follows (Eqn. 1):
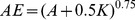
(1)where *A* is the number of adults (>14 years) in the household and *K* the number of children (0–14 years) [28].

The equity of healthcare financing was measured using the Kakwani index (KI), with KI defined as twice the area between the payment concentration curve and Lorenz curve [28]. KI (π_K_) was calculated as:
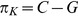
(2)where *C* is the concentration index (CI) for healthcare payments and *G* is the Gini coefficient of ATP. CI is a measure for assessing proportionality of healthcare resources within a defined population. KI was used to estimate the degree of equity in the healthcare financing system. The π_K_ value ranges between −2 to 1, with a positive number indicating progressivity (L_1_ located below L_2_), and a negative number indicating regressivity (L_1_ located above L_2_). When the concentration curve is located on the Lorenz curve, with the index equaling 0, proportionality is indicated. Progressivity (regressivity) indicates that the rich (poor) contribute a larger proportion of healthcare payments than the poor (rich) in comparison with ATP [27].

Computation of CI and the Gini coefficient requires comparison of covariance between variables and household fractional ranks according to ATP [29], [30]. The estimates of the Gini coefficient and CI may be obtained from ordinary least squares (OLS) regression of ATP and healthcare payment variables on the fractional rank in the ATP distribution (Eqn. 3) [27].
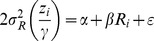
(3)where 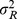 is the variance of the fractional rank, 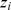 is the healthcare payment or ATP of household i, 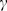 is the mean healthcare payment or ATP, 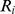 is the household fractional rank according to the ATP distribution. The OLS estimate of 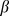 is an estimate of CI or the Gini coefficient.

As KI is the difference between CI and the Gini coefficient, both of which may be computed by the regression method above, the KI value may be computed using the following regression equation [27]:
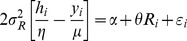
(4)where 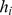 is the healthcare payment of household i, 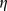 is the mean healthcare payment, 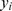 is the ATP variable, 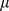 is mean ATP, 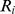 is the household fractional rank according to the ATP distribution, and 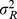 is the sample variance of the fractional rank. The OLS estimate of 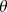 is an estimate of KI. Subsequently, overall KI of a health financing system is computed by appropriation of the weighted sum of individual KI for each source of finance, where the weight is equal to the proportion of revenue collected from each financing source.

Moreover, while KI may be used to quantify the degree of financing equity within a specific area during a specified period, it may also be used to compare levels of progressivity at different times and locations in the absence of social-economic context, due to the inclusion of economic status and health financing factors within the overall model [31]. Thus, differences of KI among different regions or time periods can be used for the assessment of regional inequality gaps and temporal variations; thus, evaluation of financing performance due to differing policies or interventions at different time intervals is permitted.

Additionally, the dominance test may be added to progressivity analyses. In order to determine if a reduction in inequity results from healthcare financing (i.e. low-income individuals contribute a smaller share of financing than the wealthy in comparison with living standards), analyses were undertaken to evaluate concentration curve dominance (i.e. lies above) with respect to the Lorenz curve for household expenditure. For dominance testing, the standard errors and differences between ordinates were computed to allow between-curve dependence where appropriate [32]. A multiple comparison approach to testing was adopted [33], with the null hypothesis defined as a lack of distinguishable difference between curves. This was tested against both dominance and crossing of curves [34]. The null hypothesis was rejected in favor of dominance in the presence of at least one significant difference between ordinates of the two curves in one direction and no significant difference in the other direction across 19 equidistant quintile points (0.05 to 0.95). The null hypothesis was rejected in favor of crossing if there was at least one significant difference in each direction [35].

## Results

Quintile-based income shares of per capita household expenditures and sources of healthcare payments (2002 and 2007) in two regions (urban and rural areas) are presented in Table 3. Financing distribution, CI, KI, and the dominance test are all employed to describe healthcare financing equity; KI variations with respect to different regions and time points are also shown (Table 3).

**Table 3:** Health care financing distribution by income quintile, concentration index (CI), and Kakwani index (KI).

Year	Area	Incomequintiles	Per capitahouseholdexpenditure	General tax	Socialhealthinsurance	Commercialhealthinsurance	OOP	Overall
2002	Urban (A)	Q1 Poorest	4.88%	5.21%	1.18%	6.53%	3.67%	0.0404
		Q2	8.57%	9.03%	4.25%	9.07%	6.82%	
		Q3	12.71%	13.12%	8.38%	10.56%	11.43%	
		Q4	19.58%	20.45%	24.71%	24.86%	15.57%	
		Q5 Richest	54.25%	52.18%	61.49%	48.97%	62.51%	
		Gini/CI	0.4839**	0.4627**	0.5809**	0.4018**	0.5714**	
		(SE)	0.0128	0.0119	0.0308	0.0651	0.0403	
		Kakwani	\	–0.0212**	0.0969**	−0.0821	0.0875*	
		(SE)	\	0.0053	0.0325	0.0668	0.0342	
		weight		0.3774	0.0462	0.0382	0.5383	
		dominance test		+	X	X	−	
	Rural (B)	Q1 Poorest	7.62%	8.23%	2.81%	4.13%	6.60%	0.0325
		Q2	11.95%	12.76%	9.64%	7.94%	9.93%	
		Q3	15.98%	16.49%	27.80%	22.03%	15.08%	
		Q4	21.76%	22.06%	20.57%	28.25%	20.35%	
		Q5 Richest	42.68%	40.46%	39.19%	37.65%	48.04%	
		Gini/CI	0.3470**	0.3174**	0.3753*	0.3621**	0.4219**	
		(SE)	0.0074	0.0066	0.1501	0.1158	0.0347	
		Kakwani	\	–0.0297**	0.0283	0.0151	0.0748*	
		(SE)	\	0.0049	0.1504	0.1162	0.0313	
		weight		0.3935	0.0053	0.0154	0.5858	
		dominance test		+	−	−	−	
2007	Urban (C)	Q1 Poorest	7.63%	8.26%	3.41%	7.99%	6.84%	0.0053
		Q2	12.08%	12.30%	9.41%	10.95%	12.71%	
		Q3	16.14%	16.04%	15.88%	9.83%	17.07%	
		Q4	21.99%	21.44%	23.30%	26.01%	24.99%	
		Q5 Richest	42.15%	41.95%	48.00%	45.21%	38.39%	
		Gini/CI	0.3435**	0.3338**	0.4419**	0.3934**	0.3232**	
		(SE)	0.0067	0.0079	0.0156	0.0587	0.0036	
		Kakwani	\	–0.0097**	0.0984**	0.0499	−0.0203	
		(SE)	\	0.0031	0.0148	0.0576	0.0069	
		weight		0.3334	0.1672	0.0314	0.4680	
		dominance test		+	−	none	none	
	Rural (D)	Q1 Poorest	8.73%	8.95%	20.82%	14.55%	8.33%	0.0000
		Q2	13.13%	13.33%	19.77%	16.23%	12.98%	
		Q3	16.96%	17.29%	19.34%	17.46%	15.70%	
		Q4	22.03%	22.37%	20.69%	22.41%	20.31%	
		Q5 Richest	39.15%	38.06%	19.38%	29.36%	42.68%	
		Gini/CI	0.3018**	0.2907**	–0.0101	0.1685**	0.3291**	
		(SE)	0.0052	0.0053	0.0077	0.0451	0.0236	
		Kakwani	\	–0.0112**	–0.3119**	–0.1333**	0.0273	
		(SE)	\	0.0032	0.0095	0.0452	0.0216	
		weight		0.3928	0.0113	0.0519	0.5440	
		dominance test		+	+	+	none	
Inequality difference	Δ (urban-rural)	2002 (E)		0.0085	0.0686	−0.0972	0.0127	
		dominance test		−	none	none	−	
		2007 (F)		0.0015	0.4104	0.1833	−0.0476	
		dominance test		−	−	none	none	
	Δ (2007–2002)	Urban (G)		0.0115	0.0015	0.1321	−0.1078	
		dominance test		+	+	none	+	
		Rural (H)		0.0185	−0.3403	−0.1484	−0.0475	
		dominance test		none	+	+	none	

*Significant at 0.05.

**Significant at 0.01.

X indicates rejection of the null hypothesis that curves are indistinguishable in favor of curves crossing at the 5% significance level.

None indicates failure to reject the null hypothesis that curves are indistinguishable at the 5% significance level.

+/− indicates concentration curve dominates (is dominated by) the Lorenz curve or concentration curve in one year or area and dominates (is dominated by) the other in another year or area.

**Figure 2:**
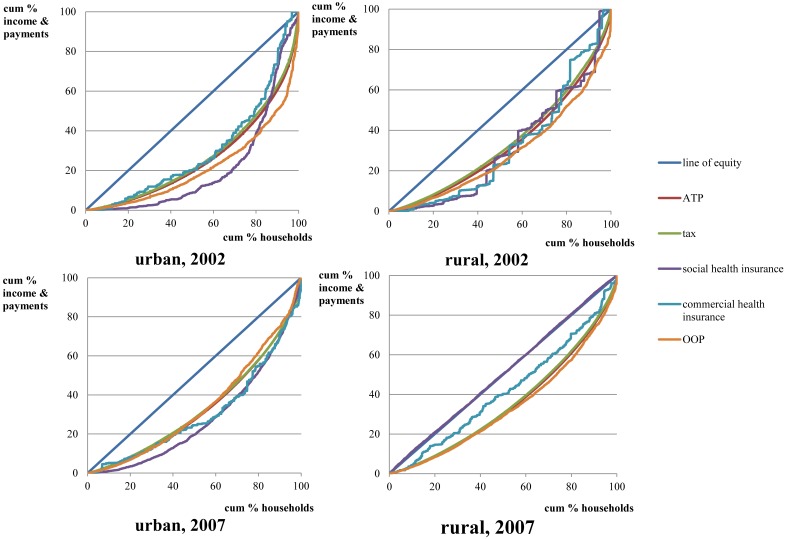
Concentration curve of healthcare payments and Lorenz curve. Actual cumulative concentration curve for healthcare payments (including general tax, social and commercial health insurances, and OOP payments) and Lorenz curves in both urban and rural areas for 2002 and 2007.

In both 2002 and 2007, all CI values were positive with one exception–rural social health insurance in 2007–indicating that the rich does not contribute a lower proportion of healthcare payments than the poor. Specifically, in both 2002 and 2007, CI values were higher in urban areas than rural areas. Furthermore, dominance testing shows that concentration curves for healthcare sources in rural areas are located above those representing urban areas. Thus, results suggest that a higher proportion of the healthcare financing burden was borne by categorically ‘rural poor’ households. Conversely, CI values for 2007 are lower than those associated with 2002 in both urban and rural areas, while the Gini coefficient was also shown to decrease over this period. Dominance testing shows that several concentration curves from 2007 are located above those from 2002. Accordingly, results indicate that a higher proportion of healthcare financing burden was borne by the poor during 2007 than that seen during 2002. In other words, results show that in terms of CI, healthcare financing became unequal over the defined study period (2002–2007) in both urban and rural areas. Additionally, the 2007 CI value associated with rural social health insurance (NCMS) was negative, albeit, not at a statistically significant level, indicating similar payment level contributions among all enrollees regardless of income (Figure 2).

KI values over the study period are also shown in Table 3. In 2002, tax financing was found to be regressive in both urban and rural areas. While the KI of social health insurance was found to be positive in cities and villages, it was not statistically significant with respect to rural areas. Accordingly, social health insurance was shown to be progressive for urban areas, and the hypothesis of proportionality cannot be excluded for rural areas. The KI for commercial health insurance was not statistically significant, indicating proportionality cannot be rejected for both urban and rural areas. Further, OOP payments were found to be progressive in both urban and rural areas. The overall Kakwani indices of 0.0404 and 0.0325 were associated with cities and villages, respectively, thus indicating a progressive healthcare financing system within both urban and rural areas.

In 2007, tax financing was found to remain categorically regressive within both urban and rural areas. Social health insurance demonstrated differing patterns with respect to cities and villages: it was progressive in cities, while found to be regressive with respect to villages. Commercial health insurance was found to be regressive in rural areas, while proportionality was not rejected for urban areas. The KI for OOP payments was not statistically significant in both urban and rural areas, and proportionality was not rejected in either case. The overall Kakwani indices were 0.0053 and 0.0000 in cities and villages, respectively, thus indicating a progressive healthcare financing system within urban areas and proportional system within rural areas; this is shown in Figure 2, with plotted Lorenz and concentration curves.

With respect to improvements and setbacks in health financing equity as indicated by measured KI values, numerous findings are presented in Table 3. Firstly, when comparing urban-rural areas (Row E), differences were typically positive during 2002, although differences pertaining to commercial health insurance were slightly negative. Moreover, during 2007 (Row F), differences were typically positive with the exception of OOP payments, which exhibited a slightly negative difference. Differences pertaining to both these exceptions were not statistically significant based upon results of dominance testing. Subsequently, urban areas were found to outperform rural areas in term of healthcare financing equity. Secondly, when comparing temporal differences, results of dominance testing indicate that an elevated financial burden is typically borne by poorer sub-populations in both urban and rural areas (Rows G–H).

## Discussion

Our study sought to provide answers to the question ‘*Does China’s health sector reform work in terms of equity in healthcare financing?’* Summarily, we conclude - “not yet”. Measured KI values of healthcare financing through taxation are close to 0 but negative, thus indicating that tax financing was slightly regressive during the period 2002–2007 in both urban and rural areas. This finding implies that the tax burden associated with healthcare financing is disproportionately borne by the low-income population. With regard to social health insurance, equity status was found to vary between cities and villages. In 2002, UWBMI was found to be progressive, while the hypothesis of proportionality cannot be excluded for CMS. While consistent efforts have been made to reform social health insurance schemes and move to universal health coverage, the progressivity of many types of insurance remains unchanged. Urban health insurance remained progressive during the study period, as evidenced by UWBMI and URBMI during 2007. Conversely, NCMS, a new type of rural health insurance, became regressive. During 2002, there was no evidence that the commercial health insurance departed significantly from proportionality in both urban and rural areas; however, it became regressive in rural areas during 2007. Typically, commercial health insurance has played a minor role in Chinese healthcare financing due to its low associated coverage [16], particularly in light of the increased preference towards social health insurance. Moreover, since initiation of China’s healthcare reform, the progressivity of OOP payment has decreased.

The aforementioned variance with respect to healthcare finance distribution in China has stemmed from several causative factors including tax categorization, health insurance schemes and care-seeking behavior due to OOP payments.

Tax financing has been shown to progressively fund healthcare systems within many high- and middle-income countries; a major reason being that the majority of these regions enforce direct taxes. However, indirect taxation comprises the majority of China’s taxation mechanisms, typically considered as being regressive tariffs and pro-wealth policies thus permitting high-income individuals to effectively transfer associated tax burdens onto lower income groups. For example, within Heilongjiang province, indirect taxation (e.g. value-added tax (VAT), sale tax, excise tax) comprised 74.00% of total taxation incomes during 2002 and 72.91% in 2007. Conversely, direct taxation accounted for 26% and 27.09% during 2002 and 2007, respectively [36]. Taxation-based financing trends exhibited little temporal change over the duration of the study period. Accordingly, high reliance on indirect taxation typically has resulted in regressive funding of the Chinese healthcare system. It is suggested that, for some indirect taxes that poorer individuals bear more burden relative to their ATP, tax reduction and abolition should be done to cope with this financing inequity, particularly in light of the recent transferal of increased financing liabilities to low-income populations [37].

Progressive trends in social health insurance were shown to differ between urban and rural areas, with individual contributions highlighted as being the primary causative factor. In categorically urban areas, the UWBMI mechanism, representing the major portion of urban compulsory health insurance, requires that all insured employees insurance premium equates to a fixed salary proportion, albeit with slight inherent variation depending on region and age. Accordingly, individual financing contributions to urban health insurance were positively correlated with income, with a higher financing burden borne by middle- and low-income groups during study period. Due to the importance placed upon universal health coverage by Chinese authorities, a large proportion of uninsured individuals, the majority of which come from low-income groups, were entitled to coverage under UWBMI and URBMI in 2007. Therefore, higher social health insurance contributions have been made by the poorer members of society than previously incurred, with accordingly decreased CI values. The Gini coefficient was shown to have decreased, resulting in a slightly higher value of KI during 2007 and increasingly progressive urban social health insurance.

With respect to rural health insurance, individual contributions were flat due to the inherent difficulties associated with determination of the socioeconomic status of rural households. With respect to CMS or NCMS, while equal premiums are required of insured households, results unexpectedly show that, in the presence of the same financing mechanism, CMS was proportional while NCMS was regressive.

China’s central authorities have defined both the aforementioned schemes as voluntary insurance programs for rural communities; CMS was ‘self-organized’ by the Production Brigade (i.e. township), with almost no responsibility borne by central or regional government under the auspices of the planned economy. While an identical premium was required of all insured individuals, enrolment was on an entirely voluntary basis. As shown (Table 2), CMS coverage in rural areas increased in concurrence with increasing household income, thus indicating that the decision to enroll in CMS was primarily based on ATP, with CMS financing found to be near proportional relative to ATP. Conversely, NCMS was re-established subsequent to the collapse of rural health insurance, traditionally considered to be a period of inaccessible healthcare. While characterized as a voluntary scheme, both central and local government objectified increased NCMS coverage, thus little effort was spared in persuasion of potential rural enrollees. As shown (Table 2), NCMS insurance rates were similar across quintiles in 2007, with all at or approaching a 90% level of coverage. This high coverage and associated flat rate have jointly contributed to imbalanced financing within NCMS as low- and middle-income groups contributed a higher proportion relative to ATP. Accordingly, the equity status of rural health insurance has deteriorated.

In the current study, OOP payments were found to be progressive during 2002, but near proportional during 2007. However, both KI and the equity status of OOP payments demand cautious interpretation. Contrary to other healthcare payments, OOP is a post-payment, accounting for the highest proportion of payments to national health accounts (NHA) [16]. In several developed countries and countries with operational public health insurance programs, low-income populations impart a higher proportion of OOP payments relative to ATP than high-income population; conversely, OOP payments are progressive in many developing countries [38]. In other words, the wealthier demographic funds a larger proportion of OOP payments, relative to ATP, than their less wealthy counterparts. However, it does not necessarily follow that these developing countries exhibit a higher level of equity in terms of OOP payments; OOP is a post-payment, therefore health care can only be administered to those in possession of adequate personal funds. In many low-income countries or regions, OOP payments do not present a barrier for the rich, even for advanced medical services and medicines, whereas the poor and lower middle-class are financially prohibited from even the most basic care [39]. In the current study, the positive KI value associated with OOP during 2002 reflects an analogous situation; due to substantial supply-based increases in healthcare costs, in concurrence with a lack of appropriate demand-based financial risk protection, a significant proportion of poorer individuals do not seek medical care due to a financial unwillingness. Conversely, wealthier individuals are not financially prohibited from paying for medical services or goods [40]. The resulting situation has concluded with the formation of progressive financing distribution. After China’s health sector reform, OOP payments became proportional, thus implying that middle- and low-income groups have started to bear an increased OOP burden. This transformation suggests that, upon enactment of health reform initiatives, including the extension of health insurance coverage and increased government health spending, financial barriers to healthcare decrease with parallel improvement in terms of patient care-seeking behaviors. Accordingly, OOP payments have increased, particularly among low-income groups, although progressivity has been shown to decrease over the same period.

The current study contains inherent limitations which require acknowledgement in order that appropriate caution is exercised when informing the policy debate surrounding healthcare reforms in China. Firstly, only a single province was examined; according, results cannot be said to be entirely representative of the characteristics of national healthcare financing. This limitation notwithstanding, our study has employed various indices for the evaluation of national policies and programs across the whole population. Therefore, our study has not considered or aligned itself with specific provincial economies or geography. To some extent, sub-national financing equity reflects the national distribution of financing.

Secondly, it is not possible to explicitly presume that renovations implemented within the healthcare financing system (e.g., extension of health insurance) have exclusively led to the observed changes in progressivity. Other causative and mitigating factors are difficult to qualify and/or quantify including geographic access, updates of medical equipment and medicines, and awareness of prevention, would have likely influenced changes pertaining to financing progressivity.

## Conclusions

The present study has shown that the overarching trend with respect to China’s healthcare financing has become increasingly unequal following health sector reforms. Tax financing was regressive during both 2002 and 2007 due to the dominance of indirect taxation within the general tax structure. Social health insurance progressivity has been shown to be strongly influenced by financing contribution mechanisms. UWBMI is progressive in cities, while NCMS is indicatively regressive due to highlighted flat-rate contributions. The progressive nature of OOP payments is unique due to its being a post-service system. Although KI decreased in both urban and rural areas, the equity status associated with the poorer sub-population has improved since extension of social health insurance programs, thus permitting access widespread to healthcare services. Therefore, effective realization of equitable healthcare financing distribution requires reduction of the type and proportion of indirect taxation on total tax revenues, particularly those focused on low-income and vulnerable groups. With regard to NCMS, levying of individual financing contributions according to rural resident’s income is suggested. Further, findings show that OOP payments still accounted for the highest proportion of total financing during 2007. Accordingly, both government healthcare spending and insurance enrollees should be increased, in concurrence with substantial updating of healthcare insurance benefit packages in order to limit the role played by direct payments.
